# Effects of Ultrasound Technology on the Qualitative Properties of Italian Extra Virgin Olive Oil

**DOI:** 10.3390/foods10112884

**Published:** 2021-11-22

**Authors:** Rossella Manganiello, Mauro Pagano, Davide Nucciarelli, Roberto Ciccoritti, Roberto Tomasone, Maria Gabriella Di Serio, Lucia Giansante, Paolo Del Re, Maurizio Servili, Gianluca Veneziani

**Affiliations:** 1 Consiglio per la Ricerca in Agricoltura e l’Analisi dell’Economia Agraria (CREA), Centro di Ricerca Ingegneria e Trasformazioni Agroalimentari, Via della Pascolare 16, Monterotondo, 00015 Rome, Italy; rossella.manganiello@crea.gov.it (R.M.); mauro.pagano@crea.gov.it (M.P.); 2 Dipartimento di Scienze Agrarie, Alimentari e Ambientali, Università degli Studi di Perugia, Via S. Costanzo, 06126 Perugia, Italy; davide.nucciarelli@studenti.unipg.it (D.N.); maurizio.servili@unipg.it (M.S.); gianluca.veneziani@unipg.it (G.V.); 3 Consiglio per la Ricerca in Agricoltura e l’Analisi dell’Economia Agraria (CREA), Centro di Ricerca Olivicoltura, Frutticoltura e Agrumicoltura, Via di Fioranello 52, 00134 Rome, Italy; roberto.ciccoritti@crea.gov.it; 4 Consiglio per la Ricerca in Agricoltura e l’Analisi dell’Economia Agraria (CREA), Centro di Ricerca Ingegneria e Trasformazioni Agroalimentari, Viale Lombardia C.da Bucceri, Cepagatti, 65012 Pescara, Italy; mariagabriella.diserio@crea.gov.it (M.G.D.S.); lucia.giansante@crea.gov.it (L.G.); paolo.delre@crea.gov.it (P.D.R.)

**Keywords:** ultrasound technology, de-stoning machine, extra virgin olive oil, qualitative profile, chemical composition

## Abstract

The development of innovative technologies in the mechanical extraction process of extra virgin olive oil can improve its quality standards through the modulation of physical, chemical and biochemical processes. Extra virgin olive oil quality and varietal differentiation are influenced by many factors, particularly the extraction. The use of ultrasound technology in the extraction process does not affect the quality, the composition, and the thermal properties of the oil, facilitating its separation from solids, and it allows the release of active compounds from the olive paste, with a positive influence on the phenolic content. In this study, the impact of ultrasound technologies was evaluated on merceological parameters, quality profile, and organoleptic features of extra virgin olive oils extracted from whole and destoned olives of the three main Italian cultivars (i.e., Peranzana, Canino, and Coratina). The parameters analyzed were influenced by both genotype and treatment, in particular, sonication did not lead to significant changes in the nutraceutical profile of the oils. The de-stoned olives were able to determine a great improvement of oil quality both for phenolic and volatile composition with a significant enhancement of health and sensory properties of the product.

## 1. Introduction

Extra virgin olive oil (EVOO), an essential ingredient of the Mediterranean diet, is a product of a huge commercial, healthy, and sensory quality, thanks to the antioxidant activity due to the presence of bioactive compounds such as mono and polyunsaturated fatty acids, phytosterols, phenolic and volatile compounds, whose concentration varies according to the technological conditions, agronomic practices, genetic and geographical origin of the cultivars [1]. Italy produces about 15% of the world’s EVOO production, after Spain, which is the world’s leading producer in terms of volumes, with an average of 20% of total European olive oil production [2]. Puglia and Calabria regions are the major oil producers, covering about 68% of Italian olive oil production. The remainder of production is in the regions of Sicily (8%), Campania (6%), Abruzzo (4%), Lazio (4%), Tuscany (3%), and Umbria (2%) [3]. In addition, with approximately 538 different olive varieties, Italy is able to express many different flavors and fragrances that make the country a unique and exclusive olive grove. Leading olive varieties include Ogliarola, Coratina, Peranzana, Cima, and Cellina in Puglia; Taggiasca and Lavagnina in Liguria; Frantoio in Tuscany; Casaliva by the Garda Lake; Moraiolo in Umbria; Coratina and Carboncella in the area of Sabina mountains (Lazio); Gentile in Abruzzo; Rotondella in Campania; Carolea in Calabria; Nocellara del Belice in Sicily and Bosana in Sardinia [3]). The development of innovative technologies in the mechanical extraction process of extra virgin olive oil can improve its quality standards through the modulation of physical, chemical and biochemical processes. Extra virgin olive oil, obtained from fruit pressing and squeezing, remains unchanged if the extraction is well performed. Quality and varietal differentiation are influenced by many factors, particularly the pedoclimatic condition and agronomical management, coupled to the extraction that, with different impacts of process variables, could affect the compositional characteristics of extra virgin olive oils from different cultivars [4]. The crushing and kneading phases are the most important. They are involved in the formation of volatile compounds through the activity of the lipoxygenase present in the fruit and the transfer of phenolic compounds and other secondary components, which are of considerable nutritional importance even if present in low concentrations. However, one of the main factors that can influence the nutritional quality of the oil is the process temperature that, following the current trend to anticipate harvest, results in high temperatures at the entrance of olives to the mill, with consequent repercussions on yield and quality of the oil. The need to innovate and enhance the different olive oil varieties present on our territory and to make Italian extra virgin olive oil increasingly competitive has led to the study of new extraction technologies that can modify the aroma and flavor of the final product or increase its antioxidant activity. In recent years, the virgin olive oil sector has attempted to convert the traditional process of extraction into a new and highly automatized one, developing innovative technologies focused primarily on improving product quality and increasing the yield of the extraction process. Upcoming technologies, such as ultrasonic waves (US), microwaves, and pulsed electric fields, are being used as alternative systems to conventional malaxation to reduce process time and improve working efficiency [5]. The use of ultrasound is a very interesting innovation, as it does not affect the quality, the composition, and the thermal properties of the oil, facilitating its separation from solids in the extraction process, and it leads to an increase in the extraction efficiency of the plants and a saving in working time, with a reduction in costs [6]. Malaxation, a crucial phase in the process of mechanical extraction of olive oil, whose efficiency depends on the rheological characteristics of the olive paste and the process parameters (i.e., time and temperature), can be significantly improved through sonication, in terms of yield and nutritional quality of EVOO. When high-power ultrasonic waves are applied to the olive paste, cavitation occurs; that is, the formation, growth, and implosion of gas bubbles during alternating pressure cycles. In this way, cellular structures break down and soluble compounds are released from the tissue of the olive plant, improving its extractability. In fact, by applying high-pressure ultrasonic waves to the olive paste, between the crushing and malaxing phases, a significant increase in extractability is achieved compared to the traditional method [7]. Sonication is a gentle, non-thermal technology of food production that allows the release of active compounds from the olive paste, with a positive influence on the phenolic content. By pumping the olive paste through the cavitation zone, ultrasonic waves are applied very uniformly to the entire compound, resulting in a qualitatively very homogeneous end product. In general, ultrasonic wave treatment increases the extraction speed and olive oil yield by milling early maturing olives, also giving olive oils enriched in bioactive compounds, particularly with regard to the phenolic fraction [8].

In addition, the introduction of heat exchangers in post-pressing, heating, or rapidly cooling the olive paste and changing the time of treatment according to the needs, can also promote the formation of all those compounds with sensory and health properties, as well as increase the yield [9].

In order to study the effects of technological processes on EVOO and table olives, the Italian Ministry of Agricultural, Food, and Forestry Policies approved and financed special research projects (Tracciabilità INFOrmativa e innovazioni di processo e di prodotto nella filiera delle OLIVe da olio e da mensa “INFOLIVA” and Miglioramento della qualità, sostenibilità e sicurezza d’uso nella DE-Amarizzazione delle OLIVe da tavola attraverso processi innovativi a scala pilota “DEAOLIVA”). In this context, different technological processes (hammer crusher without ultrasound treatment, hammer crusher and ultrasound treatment, de-stoning machine without ultrasound treatment, and de-stoning machine and ultrasound treatment) have been applied on three local cultivars to evaluate the impact of ultrasound technologies on merceological parameters, quality profile, and organoleptic features of extra virgin olive oils. Starting from these considerations, the present research aimed to assess the influence of cultivar, technological process and their interaction on the overall quality traits and nutraceutical potential of EVOO, with the final goal of providing useful information to the actors of the olive oil supply chain about innovative processes with a potential impact on the market. Furthermore, the present study describes for the first time, simultaneously, the main qualitative, nutritional, and sensorial characteristics of EVOOs derived from three autochthonous cultivars in Italy, characteristic of the Apulia region (Peranzana and Coratina) and the Lazio region (Canino).

## 2. Materials and Methods

### 2.1. Materials and Mechanical Extraction Process

Extra virgin olive oil was extracted from three of the main diffused Italian cultivars (Peranzana, Coratina, and Canino) in order to assess the impact of genetic origin and technological process on oil qualitative attributes. Specifically, olive samples of the Peranzana and Coratina cvs were purchased in the Apulia region, whereas those of the Canino cv came from Lazio, as this is an autochthonous variety of the region and grows exclusively in these specific areas. Briefly, olives were harvested from the last weeks of October to the first weeks of November and were processed within 48 h from the harvest time, at maturity indices ranging from 0.88 to 1.04. The maturity index was calculated as reported by Beltran et al. [10]. The EVOOs were extracted from approximately 200 kg of olives for each trial. The extraction plant was equipped with a dual crushing system, a hammer crusher with a 7 mm grid (Toscana Enologica Mori, Tavarnelle Val di Pesa, Florence, Italy), and a de-stoning machine (Alfa Laval S.p.A., Tavarnelle Val di Pesa, Florence, Italy). The crushed olive paste was subsequently pumped with a regular flow into a 4 kW ultrasound machine UIP 4000hdt (Hielsher Ultrasonics Gmbh, Tetlow, Germany) and thermally conditioned using an EVO-Line heat exchanger (Alfa Laval S.p.A. Tavarnelle Val di Pesa, Florence, Italy) at 18 °C. The malaxation phase was carried out at a temperature of 25 °C, with a module consisting of two malaxers with a gas controller system and a working capacity of 200 kg of olives, and the olive oil was separated from olive paste by using a two-phase decanter of 300 kg/h (Toscana Enologica Mori, Tavarnelle Val di Pesa, Florence, Italy). The final separation phase was carried out with a UVPX 305 AGT 14 centrifuge (Alfa Laval S.p.A., Tavarnelle Val di Pesa, Florence, Italy). The extraction plant was differently set up with the possibility of selecting the crushing mode and the ultrasound treatment of the olive paste by switching the ultrasound machine on or off.

The experimental tests were conducted as follows:

- Control test (CTRL) hammer crusher without ultrasound treatment;
- Test 1 (US) hammer crusher and ultrasound treatment;
- Test 2 (DS) de-stoning machine without ultrasound treatment;
- Test 3 (DS-US) de-stoning machine and ultrasound treatment.

The trials were carried out in triplicate for each cultivar.

**Figure 1:**
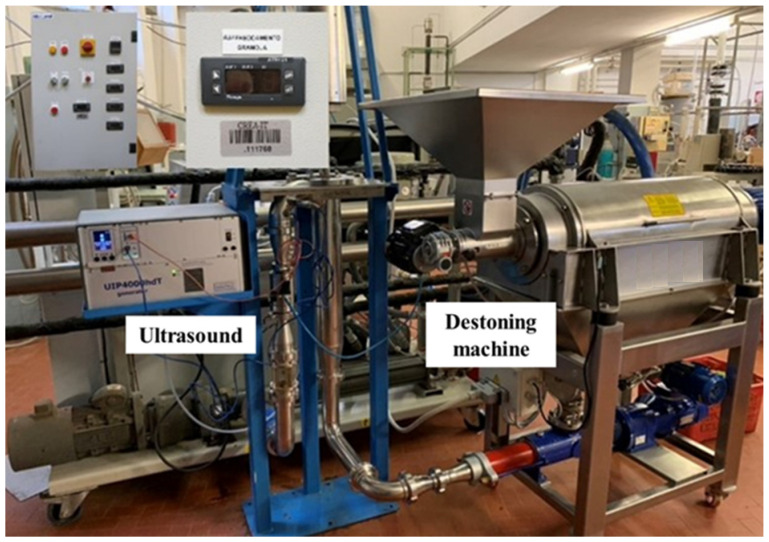
The experimental system consists of the ultrasound and destoning machine, used for the tests at the Department of Agricultural, Food, and Environmental Sciences of the University of Perugia (Italy).

The experimental equipment used for the tests is shown in Figure 1.

### 2.2. Chemical Analysis

A complete characterization of the EVOOs extracted from the three Italian cultivars was carried out by using the four different technological processes (CTRL, US, DS, and DS-US), determining the merceological indices, nutritional, and nutraceutical parameters (i.e., diglycerides, triglycerides, sterols, aliphatic and triterpene alcohols, pigments, phenolic, and volatile compounds), and sensorial attributes.

#### 2.2.1. Merceological Parameters

The EVOOs legal quality parameters, i.e., free acidity, peroxide number and UV absorption characteristics (K232, K270, and ΔK), were assessed according to the Regulation (EU) 2015/1830 [11].

In addition, the oil content of the fruits was analyzed by using a Soxhlet extractor, where 10 g of dried sample and 5 g of pumice stone were loaded into a thimble made of thick filter paper and placed inside the main compartment of the extractor. The process was carried out for six hours using hexane as an extraction solvent. The solvent was removed by using a Rotavapor R-210 rotary evaporator (Buchi Italia s.r.l, Cornaredo, Italy); subsequently, the residual oil content was detected according to the Association of Official Analytical Chemists (AOAC) method [12].

#### 2.2.2. Nutritional and Nutraceutical Analysis

The evaluation of the composition and content of diglycerides was carried out in accordance with IOC [13]. A Trace GC ULTRA gas chromatograph (Thermo Scientific, Waltham, MA, USA) fused silica capillary column (30 m × 0.32 mm ID × 0.1 μm stationary phase SE-54) was used for determinations, with a detector temperature of 350 °C and using hydrogen as the gas carrier.

Triglyceride composition was performed according to Annex XVIII of Regulation (EU) 2568/91 [14] and its subsequent amendments. The High Performance Liquid Chromatography (HPLC) analysis was conducted using a high-resolution SpectraSystem P2000 liquid chromatograph equipped with a Shodex RI SE-61 Refractive Index Detector and ERC-3312 Degasser (Thermo Fisher Scientific, Milan, Italy), a 7725 Rheodyne injector, a 10 µL sample loop. Separation on a SuperSphere 100 column (250 mm × 4.6 mm ID, 4 µm; waters) was performed at 25 °C under a constant flow rate of 0.65 mL/min with a 100% propionitrile mobile phase.

Sterol profile and alcohols content were determined according to the Regulation (EU) 2568/91 [14] and its subsequent amendments (Annexes V and XIX). The olive oil, with α-cholestanol and 1-eicosanol added as internal standards, was saponified with 2 N potassium hydroxide in ethanolic solution, then the unsaponifiable was extracted with ethyl ether. The fractions were separated from the extract by thin-layer chromatography on a basic gel plate, then recovered from the silica gel and transformed into trimethylsilyl ethers and analysed by a gas chromatograph HRGC 5160 Mega series (Perkin Elmer Milan, Italy) equipped with a Zebron Phenomenex ZB-5 capillary column (30 m × 0.32 mm ID × 0.25 μm film thickness). The gas chromatographic conditions were as follows: column temperature 265 °C; hydrogen was used as the carrier gas at a column head pressure of 50 kPa; split ratio 1:50 and substance amount injected into the split system 1 μL; injector and detector temperatures were 280 and 290 °C, respectively. The gas chromatographic conditions for alcoholic fractions were as follows: the initial isotherm was set at 180 °C for 8 min and then programmed at 5 °C min −1 to 265 °C and a further 15 min at 265 °C; the injector and detector temperatures were 280 and 290 °C, respectively.

Regarding the presence of pigments, the evaluation of spectrophotometric absorptions in the visible was carried out according to the Cucurachi method [15]. The visible absorptions between 400 and 500 nm were correlated to the concentration of carotenoids, meanwhile, absorptions between 500 and 700 nm were correlated to chlorophylls and their degradation and biosynthesis products (chlorophyllides, pheophytins, and pheophorbides).

The main hydrophilic phenols of EVOOs were extracted following the methods described by Selvaggini et al. [16], with some modifications reported in the next study of Taticchi et al. [17]. The methanolic extracts used to recover the phenolic compounds from EVOOs were subjected to high-performance liquid chromatography (HPLC) analysis. The quantitative and qualitative concentration of bioactive molecules were carried out using an Agilent Technologies system Mod. 1100, composed of a vacuum degasser, a quaternary pump, an autosampler, a thermostated column compartment, and detectors (DAD and FLD) and equipped with a C18 column (Spherisorb ODS-1 (250 mm × 4.6 mm) 5 μm particle size, supplied by Phase Separation Ltd. (Deeside, UK). The data were expressed as mg/kg of oil.

The detection of quantitative and qualitative concentrations of the main volatile compounds (pentanal, (E)-2-pentenal, hexanal, (E)-2-hexenal, (E,E)-2,4-hexadienal, 1-pentanol, 1-penten-3-ol, (E)-2-penten-1-ol, (Z)-2-penten-1-ol, 1-hexanol, (E)-2-hexen-1-ol, (Z)-3-hexen-1-ol, hexyl acetate (Z)-3-hexenyl acetate and 3-pentanone, 1-penten-3-one and 6-methyl-5-hepten-2-one) were evaluated using headspace-solid phase microextraction (HS-SPME) followed by gas chromatography-mass spectrometry (HS-SPME-GC/MS).

The sampling of EVOO headspace volatile compounds and the next GC/MS analysis were determined following the method described by Taticchi et al. [17] using an Agilent Technologies GC 7890B equipped with a “Multimode Injector” (MMI) 7693A (Agilent Technologies, Santa Clara, CA, USA) and a thermostated PAL3 RSI 120 autosampler equipped with a fiber conditioning module and an agitator (CTC Analytics AG, Zwingen, Switzerland). The detection system Agilent 5977B single quadrupole GC/MSD with an EI Extractor (XTR) source (Agilent Technologies, Santa Clara, CA, USA) was used to identify the different volatile molecules by comparison of their mass spectra and retention times with those of authentic reference compounds and with the spectra in the National Institute of Standards and Technology (NIST) 2014 mass spectral library.

The main aldehydes, alcohols, esters, and ketones were quantified using calibration curves for each compound by internal standard calculation, and the results were expressed in µg/kg of oil.

#### 2.2.3. Organoleptic Assessment

The sensory profile of the olive oils was performed in accordance with the relevant provisions of the Regulation (EU) 2568/91 [14] and its subsequent amendments (Annex XII) by the CREA Research Centre for Engineering and Agro-Food Processing of Pescara Panel, acknowledged by the International Olive Council (IOC) and the Italian Ministry of Agricultural, Food, and Forestry Policies (MiPAAF). Each taster smelled and tasted the oil according to the profile sheet of Annex XII and to IOC [18]. The following attributes were evaluated on a scale from 0.0 to 10.0: fruity, bitter, spicy, grass, almond, artichoke, tomato, apple, and aromatic herbs.

### 2.3. Statistical Analysis

All the statistical analyses described below were carried out on the mean of three replicates of each genotype for each treatment. The whole dataset was subjected to a two-way analysis of variance (ANOVA) considering the cultivar (G), the process (P), and their interaction (G × P) as a variance factor, followed by a post hoc Tukey test. All the statistical analyses reported in this section were carried out with the software PAleontological Statistics PAST (version 2.17v) [19].

## 3. Results and Discussion

### 3.1. General Aspects

Olive fruits were characterized by a moisture and oil content of 60.2 and 13.6%; 53.5% and 20.7; 55.9 and 14.0%, respectively for Canino, Coratina, and Peranzana cultivars. 

The results of the two-way ANOVA, reported in Table 1, showed significant effects of cultivar (G), process (P), and their interaction (P × G) for all merceological, nutritional, nutraceutical, and sensorial traits, except for some sterol and sensory attribute. However, in this work, it was not possible to evaluate only the effects of cultivars on total variances because it was not possible to have Canino samples growing in Apulia, since pedoclimatic conditions strongly limit the cultivation of this plant in that region. For this reason, the variability among the genotypes is not only due to genetic differences but also to the pedoclimatic conditions of the origin regions of the three cultivars, i.e., Apulia for Coratina and Peranzana and Lazio for Canino.

In detail, no significant influences of the process were observed for PLLn, OLLn + PLLn, stigmasterols, beta sitosterol, and cholesterol. In addition, the interaction G × P was not significant for cholesterol, campesterol, PLLn, and brassicasterol. The genotype was found to be the main variable for all data analyzed (never less than 50% of the total variance), except for D-7-Stigmastenol, total Sterols, and the sensory attributes of spicy. In all cases, the interaction (G × P) reported less influence on total variance with respect to the Y and G factors separately considered. An evident influence of the cultivar on olive oil composition was also reported in many studies [20,21,22]. Indeed, the quality of olive oil can be influenced by several factors, such as soil and climatic conditions and agronomic management of a specific geographical area, as reported by Inglese et al. [4]. Moreover, many studies highlight not only the influence of olive cultivars but also by climatic and environmental conditions, agronomic practice, and the technological process on the content of minor components in oils [23,24,25].

Numerous studies have evaluated the influence of transformation processes on the quality parameters of the oils, although few have highlighted the effects of ultrasound-assisted extraction [7]. Considering the extraction processes, hammer crusher, and ultrasound treatment, on average, showed the highest values for Peranzana and Canino cultivars, differently from Coratina that reported no significant difference between US process and US applied after destoning fruits (Table 1).

### 3.2. Merceological Parameters

The data related to the main legal quality parameters, i.e., free acidity (%), peroxide value (meq O_2_/kg), K_232_, K_270,_ and ΔK of all the trials carried out for the three cultivars on whole or de-stoned olive paste, treated with or without ultrasound, are presented in Table 2.

The values of the merchandising parameters did not show any significant alteration. The lack of effects on EVOO legal quality parameters has been confirmed by other authors, not only for the de-stoning process but also for new technologies with a high physical-chemical impact on the olive paste tissues [26,27,28,29]. The slight differences detected among the cultivars were not due to the different extraction processes but to the quality index and the different maturity of the olives.

### 3.3. Nutritional and Nutraceutical Analysis

The contents of sterols, di and triglycerides, and superior aliphatic and triterpene alcohols tended to be substantially higher in the Peranzana and Coratina cultivars (Table 3). In addition, significant differences were found among the process highlighting in general higher content of these compounds in control samples and US samples for all cultivars. In particular, all the analyzed olive oil samples obtained without destoning contained more than 1000 mg/kg of total sterols, the minimum value established by EU Regulation for olive oil as also reported by Rivera del Álamo [30]. In detail, the destoning process significantly affected the di and triglycerides and superior aliphatic and triterpene alcohols causing their decrease. Evidently, the destoning processing resulted in a minor release of these compounds owing to the less effective actions of the destoner with respect to metal crusher on the fruit epicarp, where these substances are mostly or thoroughly located [31,32].

**Figure 2:**
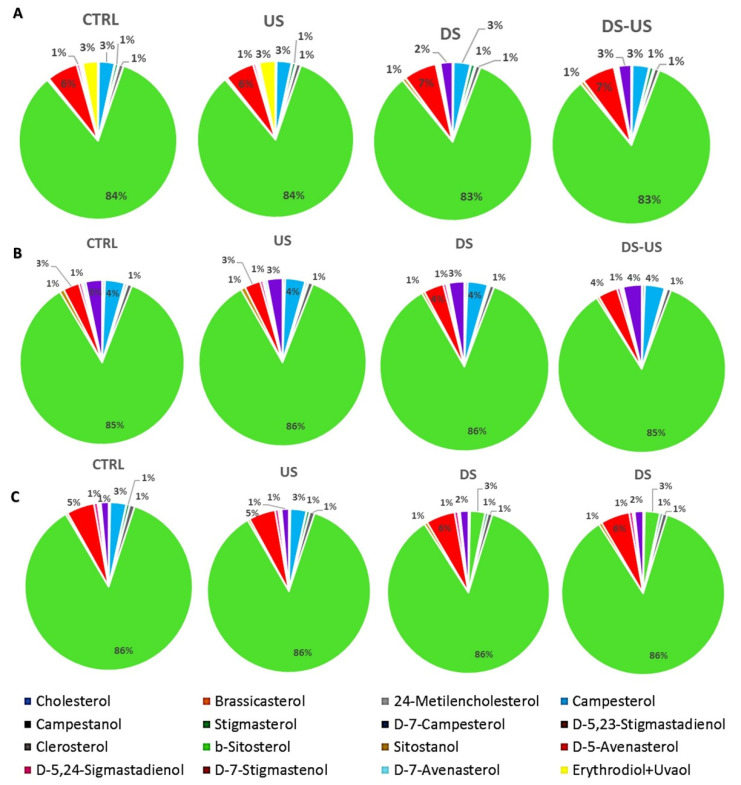
Sterol composition (%) of Peranzana (**A**), Canino (**B**) and Coratina (**C**) EVOOs, treated by the different processes (CTRL = control test, US = ultrasound test, DS = destoned test, DS-US = destoned-ultrasound test).

Gas chromatographic results of sterol percentage composition is reported in Figure 2.

As expected for virgin olive oil, the main sterols found were apparent β-sitosterol, β-sitosterol, D-5-avenasterol, and campesterol, (94.8 ± 0.5%, 87.4 ± 1.1%, 5.2 ± 1.4%, and 3.5 ± 0.4%, respectively), although in different concentrations among cultivars (Peranzana: 94.8 ± 0.2%, 86.0 ± 0.5%, 6.6 ± 0.3%, 3.4 ± 0.2%; Canino: 94.2 ± 0.1%, 88.4 ± 0.2%, 3.6 ± 0.4%, 4.0 ± 0.1%, and Coratina: 87.7 ± 0.1%, 95.4 ± 0.2%, 5.6 ± 0.2%, and 3.1 ± 0.1%) and regardless of the processing process on average no significant differences were observed. However, for the cultivar Peranzana, the content of these compounds was remarkably lower when de-stoning was applied. In addition, for campesterol, all the samples analyzed did not exceed the upper limit of the 4% set by the EU (Regulation 2568/91/EEC and later amendments). As regards the other authenticity indices established by current legislation, the apparent β-sitosterol content was lower than the legal minimum of 93% in all samples examined. All samples also exceeded the upper limits for cholesterol, brassicasterol, and D-7-stigmastenol, although these are minor components in the sterol fraction indifferently to the transformation process.

**Figure 3:**
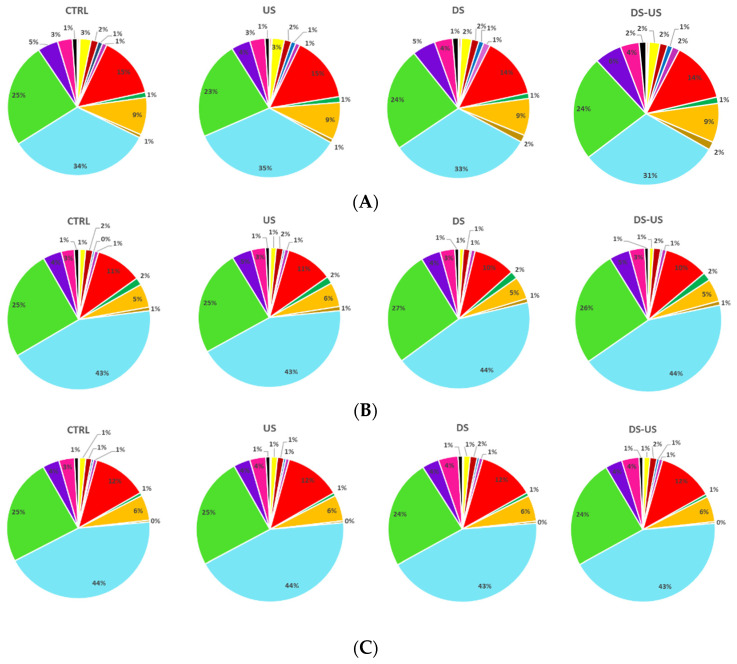
Triglyceride composition (%) of Peranzana (**A**), Canino (**B**), and Coratina (**C**) EVOOs, treated by the different processes (CTRL = control test, US = ultrasound test, DS = destoned test, DS-US = destoned-ultrasound test). LLL = trilinolein-glycerols, PLLn = palmitoyl-dilinoleoyl-glycerols, OLLn = linolenoyl-oleoyl-linoleoyl-glycerols, PLLn = dilinoleoyl-palmitoleoyl-glycerols, PoLL = dilinoleoyl-palmitoleoyl-glycerol, OOLn = dioleoyl-linolenoyl-glycerols, PoOL = palmitoleoyl-oleoyl-linoleoyl-glycerol, PoPoO = dipalmitoleoyl-oleoyl-glycerol, PPoPo = dipalmitoleoyl-palmitoyl-glycerol, PPoL = palmitoyl-palmitoleoyl-linoleoyl-glycerol, LnPP = dipalmitoyl-linoleoyl-glicerol, PLO = palmitoyl-linoleoyl-oleoyl-glycerol, POO = dioleoyl-palmitoyl-glycerol, OOO = triolein-glycerols, SLL = dilinoleoyl-stearoyl-glycerol, PoOO = dioleoyl-palmitoleoyl-glycerol, SPoL = stearoyl-palmitoleoyl-linoleoyl-glycerol, POP = dipalmitoyl-oleoyl-glycerol, PoOP = palmitoyl-palmitoleoyl-oleoyl-glycerol, SOLn = stearoyl-oleoyl-linoleoyl-glycerols, SOO = dioleoyl-stearoyl-glycerol, POS = palmitoyl-oleoyl-stearoyl-glycerol, SPoL = stearoyl-palmitoleoyl-glycerol, SLS = distearoyl-linoleoyl-glycerols.

The mean percentage content of triglyceride composition determined in the three cultivars subjected to different processing is depicted in Figure 3A–C.

The main triglycerides found were OOL + LnPP, PLP + OOO + PoPP, SOL + POO and POP (12.3 ± 1.9%, 40.0 ± 5.2%, 24.6 ± 1.0% and 4.4 ± 0.8%, respectively). The cultivar with the highest content of OOL + LnPP and POP was Peranzana (mean value across the processes: 14.4 ± 0.8%, 33.1 ± 1.6%, 23.8 ± 0.8% and 5.10 ± 0.7%, respectively), whereas for the lowest value was Canino for OOL + LnPP (10.2 ± 0.5%) and Coratina for POP (3.7 ± 0.8%). Similar high values of PLP + OOO + PoPP were found in Canino and Coratina CV (43.5 ± 0.3%), while a significantly low level was found in Peranzana (33.1 ± 1.6%). Moreover, this latter cultivar showed the lowest SOL + POO content (23.8 ± 0.7%) compared to Coratina (28.4 ± 0.3%). As reported by several authors [33,34,35], the triglyceride profile showed a high variability in relation to the investigated cultivars and could be useful to classify and characterize monovarietal oils [25].

The extraction process significantly affected triglyceride composition, but with a minor influence with respect to the cultivars. In detail, the main variation on triglycerides profiles were observed when DS was applied, however, highlighting a different effect in relation to the considered cultivars. Our results are in agreement with López-López et al. [36] who reported a strong difficulty to finding general trends in the one-by-one analysis of the triacylglycerol changes related to the transformation process, since the composition can also vary as a result of the different grade of the olives. 

**Figure 4:**
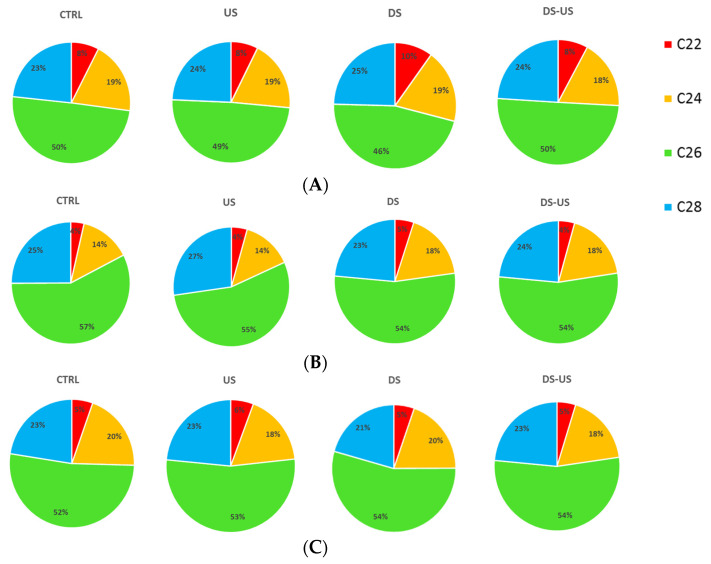
Total aliphatic alcohols (mg/kg), i.e., 1-docosanol (C22), 1-tetracosanol (C24), 1-hexacosanol (C26) and 1-octacosanol (C28), of Peranzana (**A**), Canino (**B**) and Coratina (**C**) EVOOs, treated by the different processes (CTRL = control test, US = ultrasound test, DS = destoned test, DS-US = destoned-ultrasound test).

Similar to what was reported for sterols and triglycerides, the aliphatic alcohols profile for the three cultivars and different technological processes are shown in Figure 4.

Moreover, for these compounds, the greatest differences were observed among cultivars; Peranzana showed the highest amount of all investigated alcohols (1-docosanol 5.9 ± 0.4 mg/kg; 1-tetracosanol 13.7 ± 2.1 mg/kg; 1-hexacosanol 35.5 ± 6.0 mg/kg, and 1-octacosanol 17.5 ± 2.6 mg/kg). High variability of long-chain fatty alcohols among the Italian olive oil cultivars Coratina, Leccino, Ottobratica, and Nocellara del Belice was also reported by Sicari et al. [37]. In agreement with their study, in all cultivars, the main compound found was 1-hexacosanol (mean value across cultivars and processes 31.9 ± 9.0 mg/kg), followed by 1-octacosanol (17.7 ± 4.9 mg/kg), 1-tetracosanol (10.8 ± 2.7 mg/kg) and 1-docosanol (3.7 ± 1.7 mg/kg). In general, significant differences were also observed among the extraction processes, and the highest variation was observed when DS was applied. In particular, when DS was applied, a significant decrease of 1-hexacosanol (about 3–4%) was observed with the exception of Coratina samples, where a slight increase (about 2%) was recorded.

**Figure 5:**
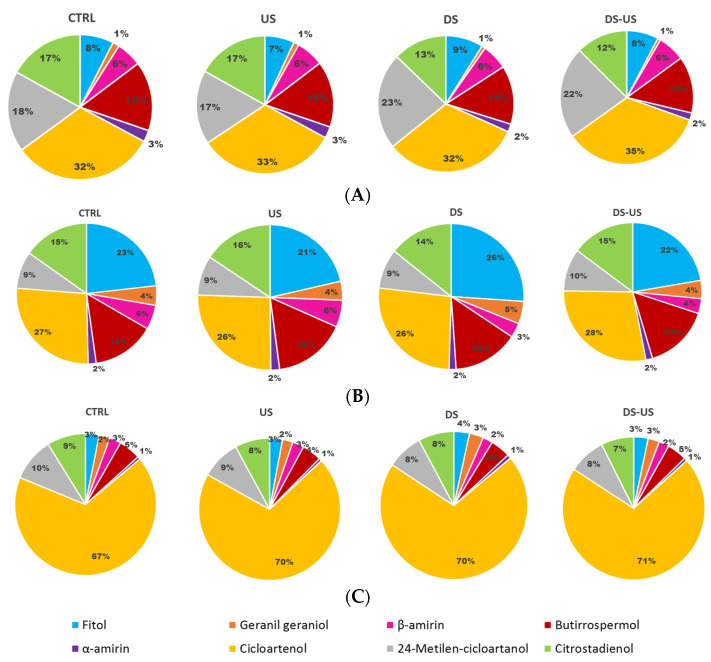
Total triterpene alcohols (mg/kg) of Peranzana (**A**), Canino (**B**), and Coratina (**C**) EVOOs, treated by the different processes (CTRL = control test, US = ultrasound test, DS = destoned test, DS-US = destoned-ultrasound test).

The main triterpene alcohols found were cicloartenol (253.8 ± 161.8 mg/kg), 24-metilen-cicloartanol (68.3 ± 31.5 mg/kg) and citrostadienol (66.8 ± 19.0 mg/kg) as shown in Figure 5. High variability among cultivars was also reported for cycloartenol, which ranged from 70% in Coratina to 27% in Canino. Similar percentage variability was found for all investigated triterpene alcohols.

As previously reported for aliphatic alcohols, the destoning process caused, on average, a significant decrease of these compounds when applied. In fact, the destoning process resulted in a lower release of these compounds due to the less effective actions of the destoner compared to metal crushing on the fruit epicarp, where these substances are more or completely located [31,32].

With regards to the evaluation of UV-Vis spectrophotometric absorptions, a significant difference between the cultivars and different processes have been observed in the ANOVA table, in relation to the EVOO pigment composition (Table 4). In particular, chlorophylls and pheophytins are responsible for the green color of the oil (UV absorption peak between 500 to 700 nm), while carotenoids, such as β-carotene and lutein, give the yellow color (UV absorption peak between 400 to 500 nm).

In detail, among the cultivars, significant differences in carotenoids, chlorophylls, and pheophytins contents were observed and the highest values in control samples were observed for Canino genotype followed by Peranzana. On the other side, the highest carotenoids/chlorophyll ratio was registered in CV Coratina. As previously reported by Cecchi et al. [38], the natural pigment contents of the oils are important quality parameters as they are involved in autoxidation and photo-oxidation mechanisms and high variability of these substances was observed. The use of ultrasound increases the pigment content of oils compared to the traditional process and de-stoning (Table 4), in agreement with the study by Clodoveo et al. [39], according to which the increase of carotenoids, chlorophyll, and tocopherol content seems to be due to the US mechanical effects, which is able to break the cell walls of plant tissues by spreading minor compounds.

**Figure 6:**
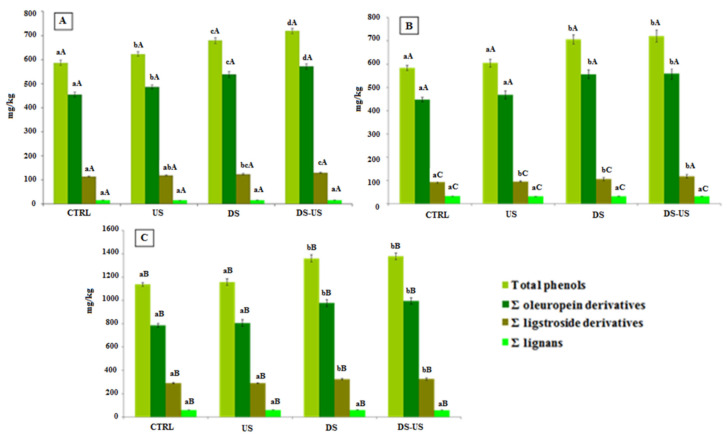
Phenolic composition (mg/kg) of Peranzana (**A**), Canino (**B**) and Coratina (**C**) EVOOs. Phenolic content was expressed as total phenols, oleuropein derivatives (sum of 3,4-DHPEA, 3,4-DHPEA-EDA, and 3,4-DHPEA-EA), ligstroside derivatives (p-HPEA, p-HPEA-EDA, and ligstroside aglycone), and lignans [sum of (+)-1-acetoxypinoresinol and (+)-pinoresinol]. The data are the mean values of the three samples analyzed in triplicate ± standard deviation. For each different cultivar, the values of each phenolic group with different lower-case letters are significantly different from one another (*p* < 0.05). For each different test, the values of each phenolic group of the three cultivars with different upper-case letters are significantly different from one another (*p* < 0.05). CTRL = control test, US = ultrasound test, DS = destoned test, DS-US = destoned-ultrasound test.

The phenolic concentration of Canino, Coratina, and Peranzana showed an improvement of total phenols of EVOOs extracted from de-stoned olives compared to the control test, with significant increases of 21, 19.7, and 15.8%, respectively (Figure 6). The effect of the destoning process on phenolic content was not influenced by the genotypes, as reported by previous studies [26,29,40]. On the contrary, Criado-Navarro et al. [41], in a recent study, showed a differentiated impact on secoiridoid content of Arbequina and Picual cultivars. From a qualitative point of view, the increases involved both oleuropein and ligstroside derivatives, while the concentration of lignans was not affected by the de-stoning process, confirming a more stable trend when influenced by the technological variabilities of the extraction process.

The use of ultrasound, both for whole and de-stoned olives, produced a slight enhancement of phenolic concentration, although the improvement was statistically significant only for the EVOOs of CV Peranzana, with an increase of 6.2% for US and 5.8% for DS-US tests compared to CTRL and DS tests, respectively. The quantitative and qualitative differences among the three cultivars were mainly due to the genetic phenolic content of the olives, with CV Coratina characterized by a high phenolic concentration, Canino and Peranzana with a medium phenolic concentration, taking into account the possible variability due to the olive ripening stage, growing area, climatic season and agronomic practices [27,42,43].

**Figure 7:**
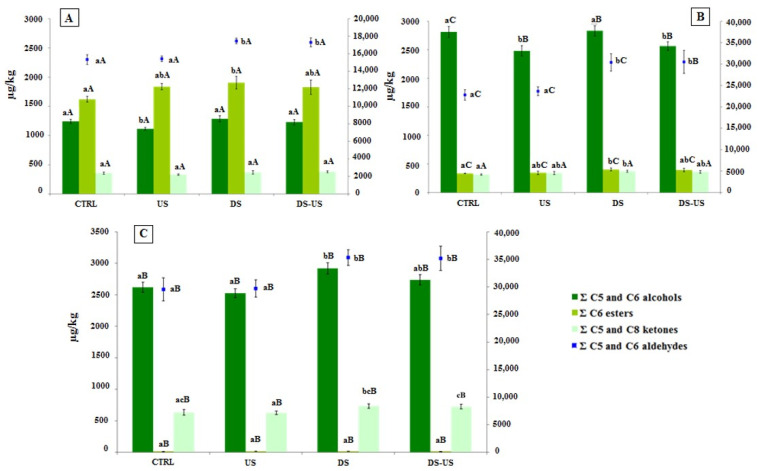
Volatile composition (μg/kg) of Peranzana (**A**), Canino (**B**) and Coratina (**C**) EVOOs. Volatile content was expressed as C5 and C6 saturated and unsaturated alcohols (sum of 1-pentanol, 1-penten-3-ol, (E)-2-penten-1-ol, (Z)-2-penten-1-ol, 1-hexanol, (Z)-2-hexen-1-ol, (E)-2-hexen-1-ol, (Z)-3-hexen-1-ol, and (E)-3-hexen-1-ol), primary axis; C6 esters (sum of hexyl acetate and (Z)-3-hexenyl acetate), primary axis; C5 and C8 ketones [3-Pentanone, 1-Penten-3-one and 6-Methyl-5-hepten-2-one], primary axis; C5 and C6 saturated and unsaturated aldehydes (sum of pentanal, (E)-2-pentenal, hexanal, (E)-2-hexenal and (E,E)-2,4-hexadienal), secondary axis. The data are the mean values of the three samples analyzed in triplicate ± standard deviation. For each different cultivar, the values of each volatile group with different lower-case letters are significantly different from one another (*p* < 0.05). For each different test, the values of each volatile group of the three cultivars with different upper-case letters are significantly different from one another (*p* < 0.05). CTRL = control test, US = ultrasound test, DS = destoned test, DS-US = destoned-ultrasound test.

The sum of the main volatile compounds (Figure 7) of EVOOs obtained by the ultrasound system showed no change in their concentration for both oils extracted from whole and de-stoned olive paste, confirming the data of previous studies [7,27]. A limited variability was highlighted for the sum of alcohols, which, however, seems to be strictly dependent on the genotype.

The olive de-stoning process showed a common increasing trend in the sum of aldehydes mainly related to the (E)-2-exenal enhancement of all EVOOs extracted from the three cultivars, when compared to the relative CTRL test. The amount of aldehydes of Canino, Coratina and Peranzana had an increase of 33.4%, 19.4% and 13.8%, respectively. The same significant increasing trend was followed by esters, even if their amount in the Coratina EVOOs was probably too low to determine a significant impact on the sensory notes of the oil. On the other hand, the sum of alcohols was not influenced by the de-stoning process, with the only exception of Coratina EVOO, which showed an increase of 11.5% compared to the CTRL test. The data confirmed the results obtained in a previous study [44] on Carolea and Ottobratica cultivars, highlighting that the higher volatile concentration of de-stoned olives compared to whole olives is independent of genotype.

### 3.4. Sensory Analysis

**Figure 8:**
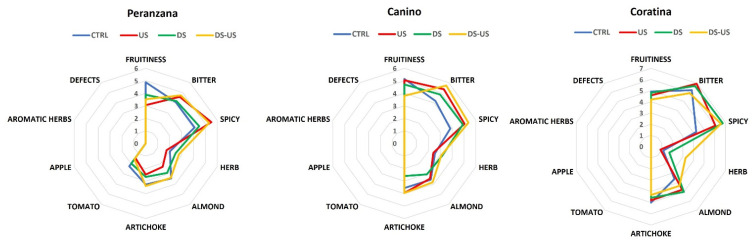
Sensorial profile of Peranzana, Coratina, and Canino EVOOs. CTRL = control test, US = ultrasound test, DS = destoned test, DS-US = destoned-ultrasound test.

The organoleptic profile of the extra virgin olive oils extracted from the three genotypes, through the different processes (i.e., hammer crusher without ultrasound treatment, hammer crusher and ultrasound treatment, de-stoning machine without ultrasound treatment, and de-stoning machine and ultrasound treatment), is reported in Figure 8.

The use of ultrasound negatively influenced the fruitiness, accentuating the bitter, spicy, and artichoke sensations, contrary to the results obtained by Jiménez et al. [45], according to which oils from US treatment were more fruity, green, pungent, and less bitter than the untreated ones. The reduction in fruitiness was probably due to an oversizing of the pressing system, which may have increased the temperature of the olive paste in the kneading trough. Only for the Coratina cultivar, the process with destoning machine and ultrasound treatment seem to favor a decrease in bitterness and an increase in herb sensation compared to the other theses. Similar results were found in the study by Clodoveo et al. [39], in which ultrasound-assisted extraction of EVOO from the CV Coratina produced a reduction in the concentration of polyphenols, improving its taste by reducing the bitter and pungent notes without affecting the fruity notes. Moreover, the increase in the “green” sensation contributes, together with the fruitiness, to enhance the positive notes of the oil taste, thanks to the higher quantity of phenolic and volatile compounds due to the removal of the seeds (which should contain enzymatic activities for the metabolism of hydroperoxides) obtained with the use of the destoning machine, giving the oil a greater sensorial score and thus a higher market value, as confirmed by the work of Amirante et al., [46].

Further studies will have to be carried out to assess the effects of kneading and ultrasound technology on fruitiness.

## 4. Conclusions

The application of ultrasound coupled with the destoning process could produce EVOO without significantly altering the quality parameters of the product, which are, however, strongly dependent on the choice of cultivars. In particular, no significant effects were observed on the merceological parameters (free acidity, peroxide value, K232, K270, and ∆K). Moreover, the main nutritional and nutraceutical variation were principally due to the destoning process and only to a minor extent to the sonication process. However, a high level of pigments (carotenoids and chlorophylls), total sterols, total aliphatic alcohols, and total triterpene alcohols were observed when ultrasound was applied, without significant variations in the percentage composition. On the contrary, the destoning process caused a decrease in many nutritional and nutraceutical parameters by altering the percentage composition of the individual compounds. In this regard, it was observed that destoned olives were able to determine a great improvement of EVOOs quality both for phenolic and volatile composition with a significant enhancement of health and sensory properties of the product. The de-stoning process was cultivar independent increasing oleuropein and ligstroside derivatives of phenolic fraction and the class of aldehydes and ester that characterize the volatile profile of the EVOO. The data confirmed the results of other studies, but further investigation on the influence of the cultivars could be carried out. The ultrasound showed a limited modification of phenolic and volatile composition, and the new technology seems to be more influenced by the genetic origin of the fruits. For these reasons, the use of ultrasound in the extraction process seems to be a valid method to increase the oil yield without altering the nutraceutical profile. Furthermore, the use of this emerging technology meets the growing consumer preference for high-quality extra virgin olive oil with health and sensory properties associated with a higher content of phenolic and volatile compounds.

Finally, EVOO obtained through ultrasound technologies showed a particular sensorial profile characterized by a bitter and spice texture, often desired for the realization of certain local culinary specialties.

## Figures and Tables

**Table 1:** Analysis of variance (ANOVA) for the parameters of visible and diglycerides (**A**), triglycerides (**B**), sterols (**C**), aliphatic and triterpene alcohols (**D**), and sensory attributes (**E**) of Peranzana, Canino, and Coratina EVOOs, extracted through different processes (i.e., control test, ultrasound test, destoned test, destoned-ultrasound test).

**A**																		
**Source**		**Degrees of Freedom**		**Mean Square**														
				**A414**		**A446**		**A474**		**A531**		**A664**		**R474/664**		**Diglycerides**		
**Sampling (S)**		3		ns		ns		ns		ns		ns		ns		ns		
**Genotype (G)**		2		5.81 ***		4.52 ***		3.06 ***		0.017 ***		0.66 ***		4.02 ***		1.96 ***		
**Process (P)**		3		0.74 ***		0.85 ***		0.71 ***		0.0003 ***		0.14 ***		2.40 ***		0.14 ***		
**G × P**		6		0.22 ***		0.22 ***		0.18 ***		0.001 ***		0.05 ***		0.31 **		0.06 ***		
**G × S**		6		0.01 ns		0.01 ns		0.01 ns		0.0002 ns		0.001 ns		0.04 ns		0.005 ns		
**P × S**		9		0.01 ns		0.01 ns		0.01 ns		0.0007 ns		0.002 ns		0.13 ns		0.009 ns		
**G × P × S**		18		0.01 ns		0.01 ns		0.01 ns		0.0009 ns		0.001 ns		0.07 ns		0.005 ns		
**Total**		47																
**B**																		
**Source**	**DF**	**Mean Square**																
		**LLL**	**OLLn + PoLL**	**PLLn**	**OLL**	**OOLn + PoOL**	**PLL + PoPoO**	**POLn + PpoPo + PPoL**	**OOL + LnPP**	**PoOO**	**SLL + PLO**	**PoOP + SpoL + SOLn + SPoPo**		**PLP + OOO + PoPP**	**SOL + POO**	**POP**	**SOO**	**POS + SLS**
**Sampling (S)**	3	ns	ns	ns	ns	ns	ns	ns	ns	ns	ns	ns		ns	ns	ns	ns	ns
**Genotype (G)**	2	0.073 ***	0.222 ***	0.034 ***	9.42 ***	0.186 **	2.355 ***	1.29 ***	73.2 ***	4.35 ***	54.95 ***	2.9 ***		575 ***	13.7 ***	7.6 ***	1.67 ***	0.68 ***
**Process (P)**	3	0.001 *	0.0001 ns	0.0002 ns	0.28 ***	0.011 ns	0.009 **	0.15 ***	1.9 ***	0.02 ***	0.14 **	0.4 ***		3.9 ***	1.9 ***	0.8 ***	1.13 ***	0.06 ***
**G × P**	6	0.001 **	0.0012 *	0.0007 ns	0.07 ***	0.025 *	0.012 **	0.13 ***	0.6 **	0.03 ***	0.09 ***	0.4 ***		3.5 ***	1.9 ***	0.7 ***	0.13 ***	0.1 ***
**G × S**	6	0.0001 ns	0.0002 ns	0.0002 ns	0.004 ns	0.009 ns	0.001 ns	0.0005 ns	0.04 ns	0.001 ns	0.01 ns	0.0002 ns		0.3 ns	0.03 ns	0.002 ns	0.007 ns	0.001 ns
**P × S**	9	0.0002 ns	0.0007 ns	0.0004 ns	0.004 ns	0.006 ns	0.001 ns	0.005 ns	0.08 ns	0.001 ns	0.01 ns	0.001 ns		0.2 ns	0.02 ns	0.006 ns	0.003 ns	0.002 ns
**G × P × S**	18	0.0001 ns	0.0003 ns	0.0004 ns	0.008 ns	0.007 ns	0.002 ns	0.004 ns	0.1 ns	0.001 ns	0.01 ns	0.001 ns		0.2 ns	0.01 ns	0.007 ns	0.01 ns	0.004 ns
**Total**	47																	
		**C**																
**Source**	**DF**	**Mean Square**																
		**Cholesterol**	**Brassicasterol**	**24-Metilencholesterol**	**Campesterol**	**Campestanol**	**Stigmasterol**	**D-7-Campesterol**	**D-5,23-Stigmastadienol**	**Clerosterol**	**β-Sitosterol**	**Sitostanol**	**D-5-Avenasterol**	**D-5,24-Sigmastadienol**	**D-7-Stigmastenol**	**D-7-Avenasterol**	**Apparent β-Sitosterol**	**Erythrodiol + Uvaol**
**Sampling (S)**	3	ns	ns	ns	ns	ns	ns	ns	ns	ns	ns	ns	ns	ns	ns	ns	ns	ns
**Genotype (G)**	2	0.07 ***	ns	0.73 ***	3.06 ***	0.003 *	0.66 ***	0.01 **	0.15 ***	0.08 ***	24.5 ***	0.20 ***	38.64 ***	0.28 ***	0.01 **	0.09 ***	5.88 ***	11.65 ***
**Process (P)**	3	0.005 **	ns	0.002 *	0.05ns	0.003 *	0.001 ns	0.003 *	0.01 **	0.02 ns	0.36 **	0.02 ***	1.35 ***	0.03 **	0.03 ***	0.03 ***	0.08 ns	0.32 *
**G × P**	6	0.003 ns	ns	0.008 ***	0.07 ***	0.002 ns	0.02 ***	0.002 **	0.001 ***	0.02 ***	0.37 ***	0.18 ***	0.05 ***	0.04 ***	0.02 ns	0.01 ***	0.17 ***	0.55 ***
**G × S**	6	0.01 ns	ns	0.0001 ns	0.0002 ns	0.0001 ns	0.001 ns	0.0001 ns	0.00003 ns	0.0001 ns	0.02 ns	0.002 ns	0.01 ns	0.001 ns	0.001 ns	0.001 ns	0.01 ns	0.003 ns
**P × S**	9	0.01ns	ns	0.0001 ns	0.0007 ns	0.0002 ns	0.001 ns	0.0001 ns	0.0001 ns	0.0001 ns	0.04 ns	0.001 ns	0.01 ns	0.003 ns	0.002 ns	0.002 ns	0.02 ns	0.05 ns
**G × P × S**	18	0.01 ns	ns	0.0001 ns	0.0009 ns	0.0001 ns	0.001 ns	0.0001 ns	0.0001 ns	0.0001 ns	0.03 ns	0.002 ns	0.01ns	0.003 ns	0.002 ns	0.002 ns	0.01 ns	0.02 ns
**Total**	47																	
		**D**																
**Source**	**DF**	**Mean Square**																
		**Fitol**	**Geranil Geraniol**	**1-Docosanol**	**1-Tetracosanol**	**1-Hexacosanol**	**1-Octacosanol**	**β-amirin**	**Butirro Spermol**		**α-amirin**		**Cicloartenol**		**24-Metilen Cicloartanol**		**Citrostadienol**	
**Sampling (S)**	3	ns	ns	ns	ns	ns	ns	ns	ns		ns		ns		ns		ns	
**Genotype (G)**	2	25,703 ***	812 ***	57.67 ***	117.06 ***	810.3 ***	267.6 ***	1391.8 ***	10,517 ***		248.6 ***		560,339 ***		21002.9 ***		3380.6 ***	
**Process (P)**	3	171.6 ***	26.7 ***	2.01 **	19.35 ***	387.8 ***	112.4 ***	360.8 ***	1222 ***		52.1 ***		8283 ***		273.4 ***		2344.4 ***	
**G × P**	6	79.8 ***	8.05 ***	0.38 ***	4.99 ***	122.6 ***	30.6 ***	50.2 ***	189.7 ***		19.3 ***		1082 ***		160.1 ***		337.6 ***	
**G × S**	6	3.04 ns	0.41 ns	0.06 ns	0.5 ns	1.6 ns	1.9 ns	0.9ns	2.2 ns		0.6 ns		59.7 ns		12.05 ns		10.2 ns	
**P × S**	9	2.1 ns	0.44 ns	0.18 ns	0.65 ns	4.3 ns	2.8 ns	2.8ns	2.8 ns		0.2 ns		17.7 ns		7.88 ns		18.1 ns	
**G × P × S**	18	3.8 ns	0.11 ns	0.05 ns	0.27 ns	1.8 ns	1.6 ns	2.6ns	5.6 ns		0.1 ns		18.5 ns		8.28 ns		19.9 ns	
**Total**	47																	
**E**																		
**Source**		**DF**	**Mean Square**															
			**FRUITINESS**		**BITTER**	**SPICY**		**HERB**	**ALMOND**	**ARTICHOKE**		**TOMATO**		**APPLE**	**AROMATIC HERBS**		**DEFECTS**	
**Sampling (S)**		3	ns		ns	ns		ns	ns	ns		ns		ns	ns		ns	
**Genotype (G)**		2	3.58 ***		17.6 ***	6.3 ***		4.52 ***	8.12 ***	12.31 ***		17.28 ***		ns	ns		ns	
**Process (P)**		3	2.73 ***		1.4 ***	6.9 ***		4.37 ***	0.29 *	1.08 *		0.19 **		ns	ns		ns	
**G × P**		6	0.79 ***		0.8 ***	0.9 **		0.74 ***	1.42 ***	0.85 ***		0.19 ***		ns	ns		ns	
**G × S**		6	0.12 ns		0.09 ns	0.04 ns		0.02 ns	0.16 ns	0.15 ns		0.01 ns		ns	ns		ns	
**P × S**		9	0.04 ns		0.1 ns	0.30 ns		0.03 ns	0.07 ns	0.22 ns		0.02 ns		ns	ns		ns	
**G × P × S**		18	0.06 ns		0.1 ns	0.16 ns		0.07 ns	0.12 ns	0.10 ns		0.02 ns		ns	ns		ns	
**Total**		47																

(**A**) ** significant at *p* < 0.01; *** significant at *p* < 0.001; bold number is the factor mostly influencing the variable. (**B**) * significant at *p* < 0.05; ** significant at *p* < 0.01; *** significant at *p* < 0.001; bold number is the factor mostly influencing the variable. P, palmitic; Po, palmitoleic; S, stearic; O, oleic; L, linoleic acids; DF, degrees of Freedom. (**C**) * significant at *p* < 0.05; ** significant at *p* < 0.01; *** significant at *p* < 0.001; bold number is the factor mostly influencing the variable. DF, Degrees of freedom. (**D**) ** significant at *p* < 0.01; *** significant at *p* < 0.001; bold number is the factor mostly influencing the variable. DF, degrees of Freedom. (**E**) * significant at *p* < 0.05; ** significant at *p* < 0.01; *** significant at *p* < 0.001; bold number is the factor mostly influencing the variable. DF, Degrees of freedom.

**Table 2:** Legal quality parameters of Peranzana, Canino, and Coratina EVOOs.

Cultivar	Process	Free Acidity (%)	Peroxide Value (meq O_2_/Kg)	K_232_	K_270_	∆ K
**Peranzana**	CTRL	0.23 ± 0.01 ^a^	5.4 ± 0.06 ^a^	1.67 ± 0.02 ^a^	0.137 ± 0.003 ^a^	−0.004 ± 0.0003 ^ab^
	US	0.25 ± 0.01 ^a^	5.6 ± 0.26 ^a^	1.64 ± 0.03 ^a^	0.129 ± 0.008 ^a^	−0.004 ± 0.0003 ^a^
	DS	0.25 ± 0.01 ^a^	5.0 ± 0.0001 ^a^	1.61 ± 0.06 ^a^	0.125 ± 0.007 ^a^	−0.002 ± 0.0005 ^bc^
	DS-US	0.25 ± 0.01 ^a^	5.8 ± 0.8 ^a^	1.62 ± 0.06 ^a^	0.132 ± 0.01 ^a^	−0.002 ± 0.001 ^c^
**Canino**	CTRL	0.22 ± 0.01 ^a^	3.4 ± 0.3 ^a^	1.695 ± 0.079 ^a^	0.162 ± 0.004 ^a^	−0.009 ± 0.001 ^a^
	US	0.23 ± 0.01 ^ab^	3.5 ± 0.4 ^a^	1.728 ± 0.026 ^a^	0.160 ± 0.001 ^a^	−0.009 ± 0.001 ^a^
	DS	0.20 ± 0.01 ^b^	3.5 ± 0.3 ^a^	1.721 ± 0.028 ^a^	0.162 ± 0.002 ^a^	−0.008 ± 0.000 ^b^
	DS-US	0.21 ± 0.01 ^ab^	3.4 ± 0.3 ^a^	1.687 ± 0.040 ^a^	0.162 ± 0.011 ^a^	−0.007 ± 0.001 ^b^
**Coratina**	CTRL	0.24 ± 0.01 ^a^	3.4 ± 0.2 ^a^	1.650 ± 0.012 ^a^	0.167 ± 0.002 ^a^	−0.001 ± 0.001 ^a^
	US	0.24 ± 0.01 ^a^	3.5 ± 0.1 ^a^	1.651 ± 0.012 ^a^	0.168 ± 0.009 ^a^	−0.001 ± 0.001 ^a^
	DS	0.23 ± 0.01 ^a^	3.4 ± 0.2 ^a^	1.651 ± 0.076 ^a^	0.168 ± 0.019 ^a^	−0.001 ± 0.001 ^a^
	DS-US	0.23 ± 0.01 ^a^	3.4 ± 0.3 ^a^	1.656 ± 0.048 ^a^	0.170 ± 0.005 ^a^	−0.002 ± 0.000 ^a^

Legend: CTRL = hammer crusher without ultrasound treatment, US = hammer crusher and ultrasound treatment, DS = de-stoning machine without ultrasound treatment, and DS-US = de-stoning machine and ultrasound treatment; different letters in the same column indicate significant differences.

**Table 3:** The mean values of total sterols, total diglycerides, total aliphatic, and triterpene alcohols, expressed in mg/kg, of the EVOOs of the three cultivars analyzed in triplicate ± standard deviation.

Cultivar	Process	Total Sterols	Total Diglycerides	Total Aliphatic Alcohols	Total Triterpene Alcohols
**Peranzana**	CTRL	1257 ± 8 ^b^	1.91 ± 0.06 ^a^	72.5 ± 1.6 ^b^	567.7 ± 12.1 ^b^
	US	1341 ± 21 ^a^	1.82 ± 0.10 ^ab^	85.9 ± 5.0 ^a^	646.1 ± 27.4 ^a^
	DS	955 ± 11 ^d^	1.60 ± 0.04 ^b^	59.4 ± 0.6 ^c^	472.6 ± 13.3 ^d^
	DS-US	990 ± 15 ^c^	1.56 ± 0.20 ^b^	72.4 ± 5.0 ^b^	507.1 ± 6.5 ^c^
**Canino**	CTRL	1280 ± 17 ^b^	1.14 ± 0.04 ^a^	74.6 ± 1.4 ^b^	451.0 ± 6.3 ^b^
	US	1406 ± 13 ^a^	1.10 ± 0.08 ^ab^	89.0 ± 6.4 ^a^	494.5 ± 13.2 ^a^
	DS	915 ± 7 ^d^	1.11 ± 0.01 ^ab^	46.8 ± 2.1 ^d^	374.7 ± 3.8 ^cd^
	DS-US	967 ± 10 ^c^	1.09 ± 0.03 ^b^	55.0 ± 1.2 ^c^	388.9 ± 11.3 ^c^
**Coratina**	CTRL	1085 ± 9 ^a^	1.75 ± 0.01 ^b^	44.0 ± 0.3 ^ab^	687.8 ± 13.1 ^b^
	US	1089 ± 5 ^a^	1.90 ± 0.01 ^a^	47.0 ± 3.6 ^a^	748.3 ± 1.7 ^a^
	DS	1017 ± 7 ^c^	1.45 ± 0.09 ^c^	42.4 ± 3.5 ^b^	594.6 ± 4.7 ^d^
	DS-US	1059 ± 29 ^b^	1.74 ± 0.03 ^b^	44.4 ± 1.3 ^a^	650.2 ± 11.2 ^c^

Legend: CTRL = hammer crusher without ultrasound treatment, US = hammer crusher, and ul-trasound treatment, DS = de-stoning machine without ultrasound treatment and DS-US = de-stoning machine and ultrasound treatment; different letters in the same column indicate significant differences.

**Table 4:** Influences of different EVOOs extraction methods on spectrophotometric UV-Vis absorbtion peaks.

Cultivar	Process	A 414	A 446	A 474	A 531	A 664	R 474/664
**Peranzana**	CTRL	1.77 ± 0.03 b	1.69 ± 0.05 b	1.45 ± 0.05 b	0.08 ± 0.00 a	0.49 ± 0.00 bc	2.95 ± 0.11 a
	US	1.97 ± 0.06 a	1.89 ± 0.06 a	1.62 ± 0.04 a	0.10 ± 0.00 a	0.58 ± 0.04 a	2.80 ± 0.11 a
	DS	1.36 ± 0.15 cd	1.14 ± 0.06 d	0.93 ± 0.13 dc	0.08 ± 0.02 a	0.36 ± 0.08 d	2.73 ± 0.78 a
	DS-US	1.55 ± 0.13 dc	1.31 ± 0.08 c	0.99 ± 0.20 cd	0.09 ± 0.02 a	0.44 ± 0.03 cb	2.25 ± 0.41 a
**Canino**	CTRL	1.99 ± 0.08 b	1.94 ± 0.08 b	1.67 ± 0.09 b	0.11 ± 0.01 b	0.51 ± 0.04 bc	3.27 ± 0.06 a
	US	2.57 ± 0.09 a	2.40 ± 0.11 a	1.99 ± 0.18 a	0.16 ± 0.01 a	0.90 ± 0.07 a	2.20 ± 0.02 d
	DS	1.44 ± 0.04 d	1.37 ± 0.04 d	1.14 ± 0.0 d	0.08 ± 0.00 d	0.38 ± 0.01 d	3.01 ± 0.01 b
	DS-US	1.80 ± 0.06 c	1.62 ± 0.00 c	1.28 ± 0.08 c	0.10 ± 0.00 c	0.57 ± 0.02 cb	2.27 ± 0.23 c
**Coratina**	CTRL	0.70 ± 0.02 d	0.72 ± 0.02 dc	0.61 ± 0.01 dc	0.04 ± 0.00 dc	0.14 ± 0.00 d	4.36 ± 0.10 a
	US	0.83 ± 0.04 ba	0.83 ± 0.03 ba	0.69 ± 0.03 ba	0.05 ± 0.01 ab	0.20 ± 0.02 b	3.49 ± 0.14 bc
	DS	0.75 ± 0.02 c	0.75 ± 0.03 cd	0.63 ± 0.02 cd	0.04 ± 0.01 cd	0.18 ± 0.01 c	3.49 ± 0.02 bc
	DS-US	0.89 ± 0.05 ab	0.87 ± 0.01 ab	0.71 ± 0.03 ab	0.05 ± 0.00 ba	0.25 ± 0.02 a	2.87 ± 0.09 d

Legend: CTRL = hammer crusher without ultrasound treatment, US = hammer crusher and ultrasound treatment, DS = de-stoning machine without ultrasound treatment and DS-US = de-stoning machine and ultrasound treatment different letters in the same column indicate significant differences.

## Data Availability

Not applicable.
